# Online Learning in Medical Student Clerkship: A Survey of Student Perceptions and Future Directions

**DOI:** 10.7759/cureus.54541

**Published:** 2024-02-20

**Authors:** Rina Patel, Susan L Bannister, Erin Degelman, Tejeswin Sharma, Tanya N Beran, Melanie Lewis, Chris Novak

**Affiliations:** 1 Postgraduate Medical Education, University of Calgary, Calgary, CAN; 2 Pediatrics, University of Calgary, Calgary, CAN; 3 Postgraduate Medical Education, University of Manitoba, Winnipeg, CAN; 4 Community Health Sciences, University of Calgary, Calgary, CAN; 5 Pediatrics, University of Alberta, Edmonton, CAN

**Keywords:** technology-enhanced education, flipped classroom, asynchronous curriculum, fourth-year medical student education, emergency remote teaching

## Abstract

Background

The coronavirus disease 2019 (COVID-19) pandemic had a major impact on medical education with clerkship students abruptly removed from clinical activities in 2020 and hastily immersed in online learning to maintain medical education. In 2022, students returned to in-person clinical experiences, but synchronous learning sessions continued online with extensive use of asynchronous online resources. This change offers a unique opportunity to gather information about students’ perspectives regarding the acceptability and effectiveness of online learning strategies. This study aims to explore the clerkship student experience with the integration of online learning and in-person learning into formalized educational sessions in clerkship.

Methodology

The authors administered an online survey to clerkship students at the Cumming School of Medicine at the University of Calgary, Canada in spring 2022. The survey consisted of primarily Likert-style questions to explore the perceived effectiveness of various online learning strategies. Results are reported as the proportion selecting “quite effective” or “extremely effective.”

Results

A total of 89 students responded to the survey (57.4% of graduating class). For synchronous online learning, case-based learning was perceived as the most effective teaching strategy (61.8%), and audience response systems were the most effective strategy for improving audience engagement (70.1%). For asynchronous online learning, interactive cases (84.9%) and student-developed online study guides (83.6%) were perceived as the most effective. Students held varying perceptions regarding how online learning impacted their well-being. When considering future clerkship curricula, the majority of clerkship students preferred a blend of in-person and online learning.

Conclusions

This study identified that most clerkship students prefer a hybrid of in-person and online learning and that ideal online learning curricula could include case-based learning, audience response systems, and a variety of asynchronous learning resources. These results can guide curriculum development and design at other medical institutions.

## Introduction

The coronavirus disease 2019 (COVID-19) pandemic had dramatic effects on medical education. This impact was particularly evident in clerkship as students were abruptly removed from clinical activities during the initial months of the pandemic response (spring 2020). This rapid move to alternative educational methods was termed “Emergency Remote Teaching” (ERT). It employed extensive use of online learning to maintain medical education [1]. Many studies have been published between 2020 and 2022 about the sudden transition to online learning for medical students with the purpose of maintaining access to education during a public health emergency [1-3]. Starting in the fall of 2021, the world started transitioning out of the initial pandemic response, and students were gradually reintroduced to in-person learning. This shift allowed educators to re-evaluate the ongoing role of online learning in medical schools [4].

In contrast to ERT, there is a longer history of planned, purposeful online learning strategies in medical education [1]. Synchronous online learning refers to live teaching sessions in which learners and teachers interact and exchange information simultaneously (e.g., video-conferenced lectures or small group sessions) [5]. Asynchronous online learning refers to students using self-directed learning from online resources at their own pace (e.g., podcasts, videos, question banks) [5]. The “flipped classroom” model is a hybrid of the two and involves asynchronous preparation followed by interactive synchronous discussion and teaching [6,7]. Online learning has been shown to be similarly effective to traditional learning strategies, but significant controversy exists about when and how to best use it [8,9].

Senior medical students in clerkship participate in clinical rotations in a range of specialties. Traditionally, medical students in clerkship (sometimes referred to as “clerks” or “clinical clerks”) have additional in-person structured teaching sessions tailored to each specialty rotation. At the time our study was conducted, clerkship-structured teaching at our institution remained mostly online despite clerks returning to fully in-person clinical rotations due to public health restrictions on large in-person group gatherings.

There may be a significant ongoing role for online learning in clerkship. At the time of conceptualizing this project, there was limited research about integrated online learning in clerkship after the initial stages of the pandemic. The experience and knowledge gained during the pandemic presents an opportunity to integrate lessons learned about online learning into future curricula.

Therefore, this study aimed to explore the clerkship student experience with online learning strategies integrated with in-person learning. The goal is to provide insights into the purposeful use of online learning in future clerkship curriculum development. This study focused on three main objectives: (1) to assess the perceived effectiveness of synchronous online learning strategies, (2) to assess the perceived effectiveness of asynchronous online learning strategies, and (3) to explore the impact of online learning on medical student well-being.

This research was previously presented as a poster presentation at the Innovations in Medical Education Conference on February 16, 2023, and an oral presentation at the International Congress of Academic Medicine on April 17, 2023.

## Materials and methods

Study design and data collection methods

We developed a cross-sectional online survey informed by best practices in the medical education literature [10]. Many survey questions were adapted from a recent program evaluation of ERT during the early stages of the COVID-19 pandemic [11]. It consisted of the following four sections per our study objectives: Demographics, Synchronous Learning, Asynchronous Learning, and Wellness. The number of questions per section varied between 3 and 12 and consisted primarily of five-point Likert-style questions. Students rated the effectiveness of four different formats of synchronous online learning as well as three different learner engagement strategies (Table 1). Additionally, students also rated the effectiveness of multiple types of asynchronous online learning resources, including interactive online cases called “Cards,” online readings, and student-made study guides.

**Table 1:** Definitions of learning terminology used in this study.

Term	Definition
Synchronous Learning	Live teaching sessions where learners and teachers interact and exchange information simultaneously (e.g., video-conferenced lectures or small group sessions)
Asynchronous Learning	Using self-directed learning to gain new information from online resources at a learner’s own pace (e.g., podcasts, videos, question banks)
Case-Based Learning	Using clinical cases and collaborative discussion to reinforce clinical knowledge, teamwork skills, and clinical skills
Flipped Classroom	A hybrid model that involves asynchronous preparation followed by synchronous discussion and teaching
Audience Response System	Online platforms used in synchronous sessions that allow for direct audience engagement with the presenter, often in the form of anonymous polls or multiple-choice questions
Didactic Lecture	A synchronous session where the teacher directly presents a topic to a group of students, usually accompanied by a visual aid (e.g., PowerPoint® presentation, whiteboard illustrations)
Breakout Rooms	A strategy used in synchronous sessions where a teacher will divide students into groups for further discussions or activities related to the teaching topic
Collaborative Documents	A strategy used in synchronous sessions where a teacher provides an online document (e.g., Google Documents) where students can anonymously and simultaneously add thoughts/ideas and collaborate on questions asked during sessions

The survey was reviewed and revised by members of the research team which included clerkship students, residents, and staff physicians. The survey was also piloted with three recently graduated students (class of 2021) using concurrent probing and revised accordingly to improve content validity. The complete survey is available as Supplemental Digital Appendix 1.

The target population for this study was medical students in the Cumming School of Medicine, University of Calgary (in Alberta, Canada), class of 2022 (n = 156). This population was selected because these students uniquely experienced pre-pandemic in-person learning, pandemic online learning, and post-pandemic hybrid learning. To be eligible for participation, an individual needed to be a clerkship student at the Cumming School of Medicine with planned graduation in spring 2022. There were no exclusion criteria if participants met the previous criteria.

The survey was distributed online via Qualtrics®. Students were asked to rate only those learning strategies with which they had first-hand experience. Information was collected anonymously, and incomplete survey responses were included in the analyses (n = 9/89, 10.1%). Recruitment for the survey occurred through two formal emails from the Undergraduate Medical Education office and posts on private class Facebook groups. As an incentive, we offered a maximum $2,000 donation to the graduating class fund if survey responses exceeded 50% of the graduating class. This survey was open from March to May 2022. The study was approved by the University of Calgary Conjoint Health Research Ethics Board (REB21-1955).

Data analysis

Data were analyzed with descriptive statistics using proportions. Gender and age differences in ratings of the effectiveness of synchronous and asynchronous learning strategies were compared using analysis of variance for gender and Pearson product-moment correlation for age. Gender and age comparisons of different synchronous and asynchronous teaching strategies could not be conducted due to wide variability in the number of students who had experience with each teaching strategy. As a total of 33 comparisons of the demographic variables and the ratings of effectiveness for various strategies were conducted, the Bonferroni correction was used to reduce the likelihood of committing a Type I error. The resulting level of significance was determined to be 0.05/33 = 0.0015. The results are, thus, considered significant if the probability values are below this value. Data analysis was conducted in SPSS version 29 (IBM Corp., Armonk, NY, USA). We used the consensus-based Checklist for Reporting of Survey Studies as a guideline for reporting the findings [12].

## Results

Participant demographics

A total of 89 participants responded to the survey (57.4% of the graduating class) and 78 participants completed all questions. Most participants identified as female (n = 55/89, 61.8%). Participant ages were 21-25 years (n = 30/89, 33.7%), 26-30 years (n = 32/89, 36.0%), 31-35 years (n = 18/89, 20.2%) and >36 years (n = 9/89, 10.1%). These characteristics differed slightly from the class of 2022 at matriculation, which identified as 59.4% male, with a mean age of 25 years [13].

Synchronous online learning strategies

Participant ratings of synchronous online teaching strategies are shown in Table 2. Case-based learning was rated as the most effective for student learning, with 61.7% (50/81) of students rating it as quite or extremely effective. The second highest rating of quite or extremely effective was for online simulation (n = 34/72, 47.2%), followed by didactic lectures (n = 32/81, 39.5%). The lowest rating was for flipped classroom (n = 28/71, 39.4%). For student engagement strategies, audience response systems (e.g., polling) were viewed as the most effective, with 56/79 (70.9%) students rating them as quite or extremely effective. Breakout rooms (25/78, 32.1%) and collaborative documents (19/71, 26.8%) were viewed less favorably. There were no gender differences in these ratings (p > 0.0015). In terms of age, only one strategy was significant whereby younger students were likely to consider audience response systems to be effective for engagement (r = -0.41, p < 0.0015).

**Table 2:** Perceived effectiveness of synchronous online learning strategies.

Education strategy	Number of complete responses	Perceived effectiveness, n (valid percent)				
		Not at all effective	Slightly effective	Moderately effective	Quite effective	Extremely effective
Teaching strategy						
Case-Based Learning	81	2 (2.5%)	7 (8.6%)	22 (27.2%)	37 (45.7%)	13 (16.0%)
Didactic Lectures	81	1 (1.2%)	20 (24.7%)	28 (34.6%)	28 (34.6%)	4 (4.9%)
Flipped Classroom	71	9 (12.7%)	14 (19.7%)	20 (28.2%)	20 (28.2%)	8 (11.3%)
Online Simulation	72	3 (4.2%)	14 (19.4%)	21 (29.2%)	26 (36.1%)	8 (11.1%)
Engagement strategy						
Breakout Rooms	78	11 (14.1%)	21 (26.9%)	21 (26.9%)	21 (26.9%)	4 (5.1%)
Audience Response Systems	79	2 (2.5%)	7 (8.9%)	14 (17.7%)	32 (40.5%)	24 (30.4%)
Collaborative Documents	71	11 (15.5%)	25 (35.2%)	16 (22.5%)	14 (19.5%)	5 (7.0%)

When asked how many hours per week of synchronous teaching sessions participants would prefer in clerkship, the most frequent response was three to four hours per week (n = 43/81, 53.1%). Some students preferred five to eight hours per week (n = 14/81, 17.3%), and others preferred less at one to two hours per week (n = 21/81, 25.9%) or none at all (n = 3/81, 3.7%). When asked if it was easier to ask questions in online learning sessions than in person, there was no clear preference between either agreed or strongly agreed (29%) and disagreed or strongly disagreed (37%).

Asynchronous online learning resources

Table 3 summarizes the ratings of learning effectiveness for various asynchronous online learning resources. The most highly rated resources were Cards, with 62/73 (84.9%) students rating this strategy as quite or extremely effective, followed by student-made study guides (n = 51/61, 83.6%). The lowest rated resource was recorded lectures (n = 26/47, 55.3%). There were no gender or age differences in reported learning effectiveness (p > 0.0015). The majority of students appreciated protected time to study using online asynchronous learning resources, with 70/78 (89.7%) selecting agree or strongly agree.

**Table 3:** Perceived effectiveness of asynchronous online learning resources.

Educational resource	Number of complete responses	Perceived effectiveness, n (valid percent)				
		Not at all effective	Slightly effective	Moderately effective	Quite effective	Extremely effective
Cards	73	0 (0.0%)	3 (4.1%)	8 (11.0%)	26 (35.6%)	36 (49.3%)
Online Readings	68	0 (0.0%)	2 (2.9%)	15 (22.1%)	29 (42.6%)	22 (32.4%)
Podcasts	61	1 (1.6%)	3 (4.9%)	18 (29.5%)	28 (45.9%)	11 (18.0%)
Question Banks	35	0 (0.0%)	2 (5.7%)	11 (31.4%)	11 (31.4%)	11 (31.4%)
Recorded Lectures	47	0 (0.0%)	3 (6.4%)	18 (38.3%)	18 (38.3%)	8 (17.0%)
Student-Made Study Guides	61	0 (0.0%)	1 (1.6%)	9 (14.8%)	31 (50.8%)	20 (32.8%)
Videos	42	0 (0.0%)	1 (2.4%)	11 (26.2%)	22 (52.4%)	8 (19.0%)

Given the wide availability of asynchronous resources online, the survey explored participants’ use of resources provided by the clerkship and those discovered by students themselves. Most students used a mix of both, with 3/78 (3.8%) using only clerkship resources, 17 (21.8%) using mostly clerkship resources with few other resources, 31 (39.7%) reporting similar use of clerkship and other resources, 25 (32.1%) using mostly other resources with some clerkship resources, and 1 (1.3%) using only other resources. Many students reported that resources they discovered online were more useful than those provided by the clerkship rotation, with 38/78 (48.7%) selecting agree or strongly agree, 36 (46.2%) selecting neither agree or disagree, and 4 (5.1%) selecting disagree or strongly disagree. Frequently used asynchronous resources as indicated by students included UpToDate®, Toronto Notes (a peer-reviewed study guide developed by medical students at the University of Toronto, Canada that succinctly discusses major medical topics in point form), Osmosis, Anki, free open-access medical education podcasts, YouTube, and Calgary Guide (a peer-reviewed website that provides mind maps of a variety of medical topics developed by medical students and physicians).

Student well-being

Students reported a range of opinions when asked if online learning had increased their overall sense of wellness, with 29/78 (37.1%) selecting agree or strongly agree, and 23 (29.5%) selecting disagree or strongly disagree. Students reported that synchronous online learning sessions were more convenient to attend than in-person sessions, with 68/81 (84.0%) selecting agree/strongly agree. They also felt online learning was less disruptive to their clinical rotations, with 60/81 (74.1%) selecting agree or strongly agree.

Many students felt that the shift to online learning had increased their sense of social isolation, with 53/78 (67.9%) selecting agree or strongly agree. Most students selected disagree or strongly disagree when asked if online sessions provided a meaningful opportunity to connect with classmates (54/78, 69.2%) or instructors (51/78, 65.4%).

Overall teaching preferences

Participants were asked what model of learning they would prefer in clerkship if public health measures allowed. We found that 11/78 (14.1%) preferred all in-person learning, 22 (28.2%) preferred mostly in-person with some online learning, 28 (35.9%) preferred a similar amount of in-person and online learning, 14 (17.9%) preferred mostly online learning with some in-person learning, and 3 (3.8%) preferred all online learning. When asked if they were more likely to meet their learning objectives through online learning compared to in-person learning, 21/78 (26.9%) selected agree or strongly agree compared to 23 (29.5%) who selected disagree or strongly disagree.

## Discussion

This cross-sectional, single-institution, survey-based study explored a unique group of students who experienced pre-pandemic, pandemic, and initial post-pandemic curriculum. To address this study’s original objectives, (1) students perceived case-based learning as an effective learning strategy, especially when paired with audience response systems to enhance engagement; (2) students perceived Cards, or interactive online case-based questions, as an effective asynchronous learning strategy; and (3) students described that online learning, while convenient compared to in-person learning, also increases feelings of isolation. Overall, it revealed that students prefer a mix of online and in-person structured teaching during clerkship.

Synchronous online learning

Our study highlights specific synchronous online learning strategies that students identified as more and less effective. Case-based learning was rated as the most effective learning synchronous strategy in this study. This result is similar to a recent systematic review, which showed that both students and instructors enjoy this type of instruction, perhaps because it promotes motivation for learning [14]. Motivation for learning was particularly enhanced by the interactive component from both the learner and instructor involved in case-based learning. Furthermore, McLean’s review of case-based learning in medical education indicates that when implemented with adequate participation from both educators and students, case-based learning can span all four stages of Kirkpatrick’s model of learning that include enabling a positive reaction, acquiring knowledge, changing behaviors, and, finally, affecting practice behaviors [15,16]. There are multiple published examples of case-based learning being successfully implemented online, including during the COVID-19 pandemic [14,17].

Audience response systems, such as Poll Everywhere (https://www.polleverywhere.com/) and Kahoot! quizzes (https://kahoot.com/academy/study/), were described in our study as an effective strategy for maintaining participation during synchronous sessions. Audience response systems have been shown to increase knowledge retention through retrieval practice and a previous review of audience response systems in post-secondary education shows that they improve student engagement, increase attendance, stimulate discussion, improve learning performance, and provide feedback to teachers [18-20].

Potential challenges for online synchronous learning include teleconferencing fatigue; research examining this shows that effective audience engagement may help circumvent fatigue [3]. As such, audience response systems or other audience engagement strategies should be regularly implemented within synchronous online sessions. Additionally, our results suggest that students prefer limiting synchronous online learning to three to four hours per week, with the remaining time recommended for in-person activities or clinical experiences.

Asynchronous online learning

Students in this study identified a variety of effective online asynchronous learning strategies, with the most effective identified as Cards. Cards is an open-access electronic resource developed by educators at the University of Calgary (https://cards.ucalgary.ca/). It uses automatic item generation to create a large number of multiple-choice questions to reflect different clinical presentations [21]. Cards uses a variety of concepts to develop clinical decision-making skills, including identifying key features from patient cases to form a mental abstraction or problem representation, creating illness scripts, and using knowledge organization, storage, and retrieval theories [22]. The Cards website currently has a library of over 350,000 cases [23].

Student-made study guides were also described as particularly effective. These guides include peer-reviewed student-made resources such as Toronto Notes (https://torontonotes.ca/) and Calgary Guide (https://calgaryguide.ucalgary.ca/) which provide short written and/or graphical summaries of major topics. As discussed in a review examining study guides in medical education, peer-reviewed study guides provide structure to help with the identification of specific learning gaps and the development of further learning objectives [24]. Study guides are a form of independent learning with the impression of receiving guidance from an experienced tutor [24].

We were surprised that some students found resources discovered online to be more valuable than resources provided by the program. We propose the following contributing factors: learners value autonomy in self-directed learning, online resources are perceived as high quality, and word-of-mouth near-peer recommendation is powerful in spreading asynchronous online resources. Perhaps leaders in medical education could identify what resources their students are using, verify their quality and accuracy, and curate them to provide an array of resources tailored to course objectives.

Interestingly, students in this study did not rate flipped classroom sessions as particularly effective. There is widespread implementation of the flipped classroom type of pedagogy in medical education. The meta-analysis by Chen et al. on flipped classroom learning found that it is associated with high academic outcomes for theoretical knowledge compared to traditional didactic learning models [7]. Conversely, there was no advantage for flipped classroom with regards to practical application such as in oral clinical examinations [7]. Another systematic review of student perceptions of the flipped classroom model described that students enjoy the model and find it increases learning motivation and engagement [25]. The discrepancies between our study and other studies may lie in the potential differences in how flipped classroom teaching is delivered at our institution or the lack of clarity around the definition of flipped classroom teaching for participants completing the survey. Additionally, it is possible that flipped classroom is less favorable in comparison to the variety of other learning strategies that students are provided.

Wellness

Many studies show that medical student well-being declined during the early stages of the COVID-19 pandemic; however, it is unclear whether online learning was a primary driver or contributor to this phenomenon [26-29]. A benefit described by students relating to well-being was the convenience of online synchronous sessions. Clerkship involves participation in clinical learning, which makes this portion of training distinct from earlier years in medical training (pre-clerkship) where learning is primarily through traditional classroom methods. Clerkship rotations frequently use a distributed medical education model with students throughout different hospitals, clinics, and communities [30]. Distributed models of education are well-suited to online learning strategies to provide equivalent experiences to students at different learning sites. Students described that having online synchronous sessions was less disruptive to their clinical time, thereby encouraging participation from any site and efficient return to clinical duties after sessions. Further literature supports that this convenience may allow for more meaningful clinical experience and professional identity formation, both of which have been demonstrated to be pillars of clerkship learning [31].

Students described increasing social isolation with online learning. This phenomenon has been widely discussed [26,27]. McInnerney described the inability to freely and immediately communicate with peers and instructors as the main factor in feeling isolated with online learning [32]. This study describes that the utilization of synchronous communication before and during teaching sessions might be used in online learning to ameliorate feelings of isolation and form a sense of community [32].

The Association of Faculties of Medicine of Canada, which represents all faculties of medicine in Canada, has recently endorsed the importance of health-promoting learning environments and has adopted the Okanagan Charter (2015) [33]. This charter was developed by a variety of stakeholders from more than 45 countries and the World Health Organization [34]. It provides a framework for how we can continuously evaluate our work on well-being within medical education. Therefore, it is important that we assess the impact of online learning strategies on student well-being [35].

This study has several strengths. We surveyed a graduating class of students with a unique experience over three years, including starting pre-clerkship in-person, an abrupt transition to ERT, and a transition back to in-person clinical learning. These students experienced a range of online learning strategies and have valuable insights. We had a high response rate of 57% of the graduating class, which could be attributed to student interest in this topic, as well as an effective incentive structure. We also may be the first group to survey clerkship students about online learning after returning to full in-person clinical activities.

Regarding limitations, the nonprobability sample was obtained at a single site and may not reflect the experience of students at other institutions. Our survey was focused on student reactions to online learning strategies, which are the first level of the Kirkpatrick model of program evaluation [16]. We acknowledge that the perceived effectiveness of learning from students might not reflect actual learning outcomes. For example, we did not investigate the impact on learning outcomes by assessing exam scores. Although this study focused on students’ perceptions of effective online learning, it does not include other stakeholders’ perspectives regarding online learning, such as educators. Finally, although we did develop the survey based on previous program evaluations and conducted a pilot test of our survey questions, we have only limited content validity of our scores.

Next steps include examining the effects of online learning on higher-level learning outcomes. Additionally, it is important to explore the effectiveness of current hybrid models of learning compared to previous in-person models.

## Conclusions

This study investigated the effectiveness of online teaching strategies to further inform implementation into the typical post-pandemic clerkship curriculum. The main findings reveal that students would like to have a hybrid model of learning with both online and in-person approaches within formal curricula. Further, effective online learning curricula can include case-based learning, audience response systems, and a variety of asynchronous learning resources. Interestingly, this study also uncovered that students prefer self-discovered asynchronous resources more than program-provided resources. Finally, the use of online learning should be monitored to assess the effects on learner well-being. Understanding these preferences for online learning may help to guide future curriculum development at other medical institutions.

## Appendices

Supplemental Digital Appendix 1: Survey Questions

**Table 4:** Survey questions.

Introduction	
The purpose of this research project is to explore the experience of medical students in clerkship with online learning. This includes group/synchronous online learning (e.g., Zoom lectures or small groups) and independent/asynchronous online learning (e.g., videos, podcasts, online reading, question banks, Cards etc.). This also includes resources provided by the Cumming School of Medicine and resources independently discovered for self-directed learning. Results will be anonymized and reported in a cumulative format. Your decision to complete and return this survey will be interpreted as an indication of your agreement to participate. For further information, please see the attached implied consent document.	
Demographics	
1. What gender do you most identify with?	Female; Male; Non-Binary; Other; Do not want to disclose
2. How old are you?	<=20; 21–25; 26–30; 31–35; 36–40; >=41; Do not want to disclose
3. Which core clerkship rotations have you completed at least 2 weeks of?	Family Medicine; Internal Medicine; Pediatrics; Obstetrics and Gynecology; Psychiatry; Surgery; Emergency Medicine; Anesthesia; UCLIC
Group (Synchronous) Online Learning	
Group (synchronous) online learning refers to live sessions where the student and instructor interact simultaneously. This could include video-conferenced lectures or small group sessions.	
1. How effective were the group (synchronous) online learning sessions in each of the following clerkships? (Rate all that apply): a) Family Medicine; b) Internal Medicine; c) Pediatrics; d) Obstetrics and Gynecology; e) Psychiatry; f) Surgery; g) Emergency Medicine; h) Anesthesia; i) UCLIC	1 - Not at all effective; 2 - Slightly effective; 3 - Moderately effective; 4 - Quite Effective; 5 - Extremely effective; N/A
2. How effective were the following group (synchronous) online learning strategies for learning? If you don’t have experience with the strategy, please choose N/A: a) Didactic Lectures (Instructor teaches information with minimal interaction); b) Case-Based Learning (Instructor helps students work through cases with significant interaction); c) Flipped Classroom (Students prepare with resources prior to the session, and then instructor helps student work in small groups with significant interaction)	1 - Not at all effective; 2 - Slightly effective; 3 - Moderately effective; 4 - Quite Effective; 5 - Extremely effective; N/A
3. How effective were the following teaching techniques for group (synchronous) online learning sessions at improving your engagement with learning? If you don’t have experience with the strategy, please choose N/A: a) Breakout rooms; b) Audience response systems (e.g., Poll Everywhere, Zoom Polls, Kahoot); c) Collaborative documents (e.g., Google Documents, Padlet, Jamboard)	1 - Not at all effective; 2 - Slightly effective; 3 - Moderately effective; 4 - Quite Effective; 5 - Extremely effective; N/A
4. Do you agree/disagree with the following statements about group (synchronous) online learning resources? a) Online learning sessions are more convenient to attend than in-person sessions; b) Online learning sessions are less disruptive to my clinical rotations than in-person sessions; c) I am able to ask questions during online learning sessions	1 - Strongly disagree; 2 - Disagree; 3 - Neither agree nor disagree; 4 - Agree; 5 - Strongly agree
5. How many hours per week of group (synchronous) online learning sessions would you prefer if a clerkship rotation?	None; 1 hour; 2 hours; 3 hours; 4 hours; >4 hours
6. Please indicate the amount of preparation you typically completed prior to the teaching sessions each week for each rotation:	I did not prepare; 0–30 minutes; 31–60 minutes; 61–90 minutes; 90 minutes or more; Preparation was not required
Independent (Asynchronous) Online Learning	
Independent (asynchronous) online learning refers to resources that a learner completes independently at their own pace. These could include podcasts, videos, recorded lectures, online reading, Cards, or question banks.	
1. Which of the following independent (asynchronous) types of online learning resources did you use in clerkship?	Podcasts; Videos; Recorded Lectures; Online Readings; Cards; Question Banks; Self-Directed Modules
2. How effective were the following independent (asynchronous) types of online learning resources in clerkship? (*Only include items that the students said that they used): a) Podcasts; b) Videos; c) Recorded Lectures; d) Online Readings; e) Cards; f) Question Banks; g) Self-Directed Modules	1 - Not at all effective; 2 - Slightly effective; 3 - Moderately effective; 4 - Quite Effective; 5 - Extremely effective; N/A
3. Do you primarily use the independent (asynchronous) online learning resources provided by the clerkship or other resources that you self-identify online?	Only Clerkship Resources; Mostly Clerkship Resources, Few Other Resources; Similar use of Clerkship Resources and Other Resources; Mostly Other Resources, Some Clerkship Resources; Only Other Resources
4. How effective were the independent (asynchronous) online learning resources in each of the following clerkships? (Rate all that apply) (*Skip question if they did not use them): a) Family Medicine; b) Internal Medicine; c) Pediatrics; d) Obstetrics and Gynecology; e) Psychiatry; f) Surgery; g) Emergency Medicine; h) Anesthesia; i) UCLIC	1 - Not at all effective; 2 - Slightly effective; 3 - Moderately effective; 4 - Quite Effective; 5 - Extremely effective
5. Beyond the resources provided by the clerkship rotation, what independent (asynchronous) online learning resources have you utilized for your learning?	Podcasts - If so, name which; Videos - If so, name which; Recorded Lectures - If so, name which; Online Readings (Ex. UpToDate) - If so, name which; Question Banks - If so, name which; Self-Directed Modules - If so, name which; Student-Made Study Guides - If so, name which; Other - If so, name which; I don’t use any additional resources
6. Do you agree/disagree with the following statements about independent (asynchronous) online learning resources? a) I have enough time to study using online learning resources; b) I would appreciate protected time to study using online learning resources; c) Resources I have found online are more useful than the resources provided by the clerkship.	1 - Strongly disagree; 2 - Disagree; 3 - Neither agree nor disagree; 4 - Agree; 5 - Strongly agree
Student Experience and Wellness	
1. Do you agree/disagree with the following statements about online learning and wellness? a) Online learning has increased my overall sense of wellness; b) The shift to online learning in clerkship has increased my sense of social isolation; c) I feel like I have a meaningful opportunity to connect with my classmates during group (synchronous) teaching sessions; d) I feel like I have a meaningful opportunity to connect with my instructors during group (synchronous) teaching sessions; e) If public health measures allow, I would prefer to return to all in-person learning; f) I am more likely to meet my learning objectives through online learning compared to in-person learning	1 - Strongly disagree; 2 - Disagree; 3 - Neither agree nor disagree; 4 - Agree; 5 - Strongly agree
2. Is there anything you would like to share?	Free text
